# Concurrent JAK2-Positive Myeloproliferative Disorder and Chronic Myelogenous Leukemia: A Novel Entity? A Case Report With Review of the Literature

**DOI:** 10.1177/2324709619832322

**Published:** 2019-02-25

**Authors:** Gilbert Bader, Bernard Dreiling

**Affiliations:** 1 University of Mississippi Medical Center, Jackson, MS, USA; 2 GV Montgomery VA Medical Center, Jackson, MS, USA

**Keywords:** myeloproliferative disorder, chronic myelogenous leukemia, JAK2 mutation, BCR-ABL translocation

## Abstract

JAK2 V617F mutation and BCR-ABL translocation have been considered to be mutually exclusive. However, many cases where both hits coexisted have been reported. We have personally managed a case too. We believe this hybrid entity is underdiagnosed. Thus, we decided to shed light on this “double hit” disease to improve its diagnosis and optimize its treatment. We reviewed the English literature in PubMed since JAK2 discovery. We found 33 cases reported so far. We summarized patient characteristics and analyzed possible interactions between JAK2 and BCR-ABL clones.

## Introduction

Chronic myelogenous leukemia (CML) and Philadelphia-negative myeloproliferative disorders (MPD) are common hematologic diseases. The prevalence of CML is 1 in 17 000. Polycythemia vera (PV) with a prevalence of 44 to 57 per 100 000 and essential thrombocytosis (ET) with a prevalence of 38 to 57 per 100 000 are much more common than primary myelofibrosis (PMF), which has a prevalence of 4 to 6 per 100 000.^1^ In CML, hybrid BCR-ABL gene as a result of translocation (9,22) encodes a fusion protein that leads to activation of ABL tyrosine kinase and subsequent uncontrolled production of mature and maturing granulocytes. In Philadelphia-negative MPD, mutated Janus kinase 2 (JAK2) has increased kinase activity. It leads to proliferation of progenitors independently of cytokine stimulation. JAK2 V617F mutation is present in around 95% of PV and 50% of ET and PMF cases. BCR-ABL translocation and JAK2 V617F mutation have been considered to be mutually exclusive. However, several cases of JAK2 mutation coexisting with CML have been reported. In the pre-JAK2 era, it was difficult to confirm the coexistence of CML and Philadelphia-negative MPD due to lack of a marker for the latter. The discovery of JAK2 V617F mutation in 2005 provided the opportunity to document concomitant Philadelphia-positive and Philadelphia-negative MPD with more certainty. We used PubMed to conduct a review of cases published in English where JAK2-positive MPD and CML coexisted. Obviously, the cases were published post JAK2 discovery in 2005. We are also reporting a case that we have personally diagnosed and treated.

## Results

We found 33 cases of concomitant JAK2-positive MPD and CML. Patient characteristics are shown in Table 1. We have also personally managed a case of PMF and CML.

**Table 1.** Patient Characteristics.

Age/Sex	Diagnosis (+CML)	WBC (×10^3^)	Hb	PLT	Treatment	Course	Clone Interaction
Unknown^2^	ET	?	?	?	I	ET, CML 12 years later	?
50/Male^3^	MF	93	15	345	I	CML, MF 4 years later	BCR-ABL disappeared, JAK2 constant
43/Male^4^	PV	?	Hct 49	?	INF, HU then I	CML, PV 6 years later	BCR-ABL decreased, JAK2 constant
82/Female	PV	67	9.8	605	Radioactive P, HU, A then I	PV, CML 16 years later	?
73/Female^5^	PV	214	10.2	65	HU then I	PV, CML 15 years later	
66/Male^6^	MF	?	11.3	?	I then HU	Concomitant	?
55/Male^7^	MF	163	11.5	?	I	CML, MF 2 years later	BCR-ABL decreased, JAK2 increased
43/Male^8^	PV	100	Hct 36	?	I	PV, CML 16 years later	BCR-ABL decreased, JAK2 increased
49/Male	MF	?	?	?	I then D	CML, MF 2 years later	BCR-ABL and JAK2 decreased
64/Male^9^	?	?	?	?	I then N	Concomitant	BCR-ABL decreased, JAK2 constant
64/Male^10^	PV	15.3	24	380	I	Concomitant	BCR-ABL decrease, JAK2 increased
52/Female^11^	MF	193	10	689	HU then I	Concomitant	BCR-ABL and JAK2 decreased
58/Male^12^	MF	7.5	13.5	669	I then HU	MF, CML 4 years later	BCR-ABL decreased, JAK2 increased
67/Male^13^	MF	35	8.9	143	I then HU	Concomitant	?
32/Male	ET	35	?	907	A, HU then I	ET, CML 40 months later	BCR-ABL and JAK2 decreased
58/Female	MF	83	14.5	496	HU, INF	Concomitant	?
63/Male	PV	41	14.4	371	HU then I	PV, CML 15 years later	BCR-ABL decreased, JAK2 increased
45/Male^14^	?	57	14	266	I	Concomitant	?
39/Male^15^	PV	66	20	342	I then D then N	PV, CML 15 years later	Opposite growth
69/Male^16^	?	32.5	10.9	511	I	Concomitant	BCR-ABL and JAK2 decreased
60/Male^17^	PV	121	18.5	152	I then HU	Concomitant	BCR-ABL increased, JAK2 decreased
67/Male^18^	PV	11.9	17.2	507	INF, HU then I	CML, PV 10 years later	BCR-ABL decreased, JAK2 increased
70/Male^19^	?	11.5	15.3	950	INF then I	JAK2, 7 years after CML	?
42/Female^20^	?	350	?	498	HU then I then D	JAK2, 5 years after CML	JAK2 increased with CML treatment
53/Female	ET	36.7	12.5	983	HU then INF then I, A	CML, ET 11 years later	?
60/Female^21^	ET	23	8.3	220	I, A then D	CML, ET 2 years later	?
21/Female^22^	?	73	13.3	440	I	Concomitant	?
67/Male^23^	MF	22.6	Hct 42	652	N then D	MF, CML 3 years later	BCR-ABL decreased, JAK2 constant
77/Male^24^	?	6.2	8.7	242	?	Concomitant	?
60/Male^25^	?	30	Hct 21	324	I	Concomitant	?
61/Male^26^	PV	108	9	95	HU, I	PV, CML 7 years later	JAK2 emerged with decrease in BCR-ABL
36/Male	ET	9.4	13.8	830	HU	Concomitant	BCR-ABL increased
58/Male^27^	MF	19.7	13	285	HU, D	Concomitant	?
Our case: 75/Male	MF	23	14.3	741	HU, I	Concomitant	?

Abbreviations: A, anagrelide; CML, chronic myelogenous leukemia; D, dasatinib; ET, essential thrombocytosis; Hb, hemoglobin in g/dL; Hct, hematocrit; HU, hydroxyurea; I, imatinib; INF, interferon; JAK2, Janus kinase 2; MF, myelofibrosis; N, nilotinib; P, phosphorus; PLT, platelet count/mm^3^; PV, polycythemia vera; WBC, white blood cell count/mm^3^; ?, unknown or unclear.

**Figure 1.**
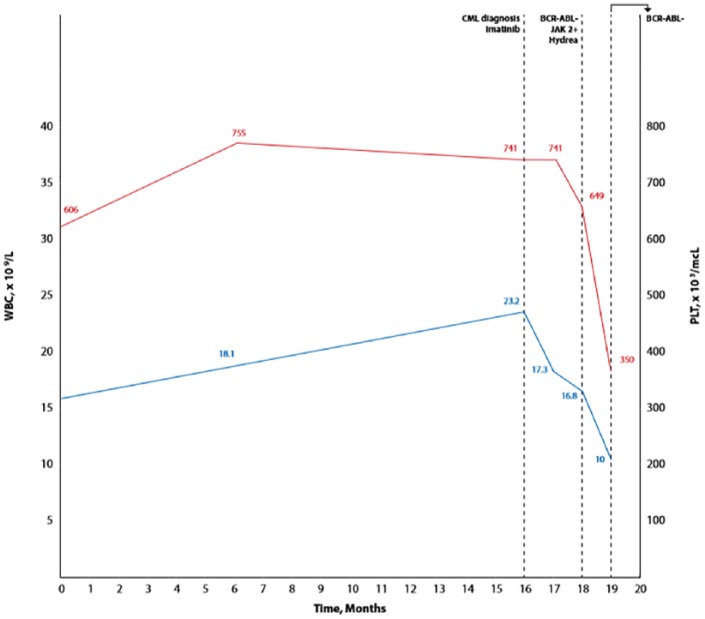
Clinical course.

The patient is a 75-year-old male who presented to our clinic in 2017 with progressive weight loss over prior 2 years, night sweats, pruritus, early satiety, and left upper quadrant pain. Physical examination was pertinent for hepatosplenomegaly. His white blood cell (WBC) count was 23 200 cells/mm^3^ with 92% neutrophils, hemoglobin 14.3 g/dL, and platelet count 741/mm^3^. He had no blasts in peripheral blood. Per chart review, we noted that he had started to develop neutrophilia and thrombocytosis in 2013 and 2014, respectively. Quantitative reverse transcription polymerase chain reaction was positive for both b2a2 and b3a2 transcripts at 2.1% and 1.2%, respectively. Imatinib was started. Eight weeks later, WBC count was 16.8 cells/mm^3^, hemoglobin 14.5 g/dL, platelet count 649/mm^3^, and reverse transcription polymerase chain reaction negative for BCR-ABL transcripts. The fact that the patient had complete molecular response without hematologic response triggered further workup. JAK2 V617F mutation was checked in peripheral blood and was positive. Erythropoietin was low at 1.3 mIU/mL. Examination of the bone marrow showed hypercellularity (90%) with myeloid/erythroid ratio of 7.7, proliferation of atypical megakaryocytes, <1% blasts, and widespread grade 2 reticulin fibrosis. No BCR-ABL translocation was detected by fluorescence in situ hybridization. DIPSS (Dynamic International Prognostic Scoring System) Plus score was 1. Myelofibrosis mutational profile analysis was not available. Hydroxyurea was added on top of imatinib (Figure 1).

Including our case, 11 patients had PMF, 10 had PV, and 5 had ET. In 8 cases, the exact nature of the myeloproliferative disorder was not clear. MPD preceded CML in 10 cases, CML preceded MPD in 9 cases, and both were diagnosed concomitantly in 15 cases. Interestingly, when MPD preceded CML, the mean time interval between the diagnoses of the 2 entities was 10.6 years compared with 5.4 years when CML preceded MPD. Interaction between JAK2 mutation and BCR-ABL transcripts levels varies. In most cases, JAK2 and BCR-ABL levels moved in opposite directions. In the remaining cases, JAK2 level decreased or remained constant while BCR-ABL level decreased.

## Discussion

BCR-ABL transcripts have been detected at very low levels in healthy persons without clinical features of CML. The amount of BCR-ABL mRNA present in healthy individuals ranged from 5 to 20 copies/5 × 10^7^ to 10^8^ WBCs.^28^ Our patient had BCR-ABL transcripts level well above this threshold. We believe he had concomitant PMF and CML. As mentioned above, JAK2 mutation and BCR-ABL translocation have been considered to be mutually exclusive. However, several cases of coexistence of both hits have been reported. The prevalence of this “dual disease” is unknown. However, we think this entity is underdiagnosed since it is rarely suspected and checked. Few studies showed surprisingly elevated proportion of this “double hit” in CML patients. In one study, 314 patients with CML were screened for the presence of JAK2 V617F mutation. The latter was present in 8 patients accounting for 2.5%.^29^ Two studies done in Karachi, Pakistan, showed proportions of CML patients harboring JAK2 V617F mutation as high as 26.7% and 44%.^30,31^

Clinically, emergence of MPD in CML patients may be mistaken for relapse or drug resistance. MPD must be suspected in CML with atypical course such as erythrocytosis or progressive or persistent thrombocytosis or neutrophilia while CML is in remission. Development of CML must be considered in cases of JAK2-positive MPD with CML-like features such as leukocytosis or progressive splenomegaly years after clinical stability.

**Figure 2.**
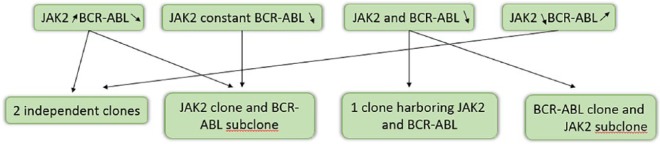
Possible JAK2 and BCR-ABL interactions and clones.

In Figure 2, we show possible interactions between JAK2 mutation and BCR-ABL transcripts levels and corresponding clonal origin. The model where BCR-ABL clone is a subclone of JAK2 one was demonstrated in a patient with CML and PV. BCR-ABL translocation and JAK2 mutation were present in most erythroid and myeloid colonies. Few colonies harbored JAK2 mutation only, and none had BCR-ABL transcripts alone.^8^ In another case, JAK2 mutation was present in CFU-GM and CFU-E compartments. However, BCR-ABL translocation was present in few CFU-GM colonies and absent in CFU-E.^6^ In 2 patients with PV and CML^32^ and a patient with PMF and CML,^23^ all hematopoietic colonies had JAK2, some had both JAK2 and BCR-ABL, and none had BCR-ABL alone. The model where JAK2 clone is a subclone of BCR-ABL one was also demonstrated. A patient with CML with positive JAK2 was treated with imatinib. Both JAK2 and BCR-ABL disappeared with treatment.^16^ The model where JAK2 and CML clones are distinct was also shown. BCR-ABL was absent in endogenous erythroid colonies formed without erythropoietin. Progressive decrease in BCR-ABL was associated with JAK2 clone expression.^10^

As mentioned above, it took around a decade in average for CML to occur after MPD. However, MPD followed CML in about 5.4 years in average. The difference between time intervals is not clear.

## Conclusion

Several cases of concomitant JAK2 MPD and CML have been reported. This hybrid disease is probably more frequent than expected because it is underdiagnosed. Providers must suspect this hybrid disease in atypical clinical course to optimize treatment and avoid transformation to acute leukemia. The World Health Organization’s MPD diagnostic criteria must probably be revised to account for the possibility of this “double hit” disease, which might be a novel clinical entity.
